# Variability in Cross-Sectional Muscle Atrophy: Insights From the Central Rectus Femoris

**DOI:** 10.7759/cureus.49097

**Published:** 2023-11-20

**Authors:** Yusuke Takahashi, Kazuki Okura

**Affiliations:** 1 Department of Rehabilitation Medicine, Akita University Hospital, Akita, JPN

**Keywords:** ultrasonography, rectus femoris, muscle thickness, muscle atrophy, central aponeurosis

## Abstract

Background and purpose: This study aimed to evaluate the non-uniformity of cross-sectional areas in atrophic muscles using the central aponeurosis (CA) as a marker for the central region of the rectus femoris (RF).

Methods: We enrolled 51 consecutively admitted patients (102 limbs) with aortic valve stenosis who were scheduled for elective surgical or catheter-based aortic valve replacement and were expected to have low physical activity-induced muscle atrophy. We obtained short-axis images of the mid-thigh using an ultrasonic diagnostic imaging system (with a 5-cm-wide probe) and measured the deviation of the central region of the rectus femoris from the body surface. Muscle thickness was measured using conventional morphological measurements on the body surface (“On Surface”) and landmarks within the ultrasonographic image (“In Images”).

Results: Displacements ≥ 1.5 cm were observed in 56 (54.9%) limbs, and displacements ≥ 2.5 cm were observed in 34 (33%) limbs. The displacements were predominantly in the medial direction and ranged from 4 cm to 1.5 cm. Among the cases in which the deviation was ≥2.5 cm, “On Surface” measurements resulted in images in which the vastus lateralis overlapped with the rectus femoris. The thickness of the rectus femoris was significantly lower with “On Surface” measurements than with “In Images” measurements (right, p < 0.001; left, p = 0.007), with a maximum difference of 10.5 mm.

Conclusions: In conclusion, it was observed that the rectus femoris at the center was often displaced medially, and the muscle thickness was thinner at the edge than at the center, showing a non-uniform morphology.

## Introduction

Skeletal muscle ultrasonography is a valuable tool to assess patients undergoing physiotherapy because of its non-invasive nature and easy repeatability. Specifically, quadriceps (Quad) muscle thickness (QMT) has emerged as an important indicator of reduced skeletal muscle mass in various clinical conditions such as aging [1], sarcopenia [2], stroke [3], critical illness [4], and cardiac failure [5].

In most cases, QMT measurements involve evaluating the cross-sectional area of the Quad muscle. Unlike ultrasonography of other body organs, QMT assessment via ultrasound is easy and reliable, and high inter- and intra-rater reliability values for its quantification have been reported. In studies focusing on healthy individuals, the intraclass correlation coefficient (ICC) for QMT measurement was reported to be 0.9 or higher [6]. However, the ICC for QMT in critically ill patients (expected to have muscle atrophy) had a moderate reported value of 0.76 [7]. These lower ICC values in atrophic muscles suggest that the health status of the population may influence the reliability of ICC measurements.

The morphology of atrophic muscles is often non-uniform, and measurement errors may occur depending on the location where muscle thickness is measured. Furthermore, in some cases, the boundaries of muscle tissue may be unclear, posing challenges in accurately identifying the muscle. These factors may contribute to variability in muscle thickness values and affect the accuracy and reliability of the results. Specifically, in cases of muscle atrophy, instances of uneven cross-sectional areas where the central portion of the rectus femoris (RF) is displaced toward the outer side, resulting in an uneven and thinner inner section, are common. This can lead to a potential underestimation of muscle thickness if the measurement points solely rely on the surface morphology of the muscle. In addition, previous muscle thickness measurements had assumed the occurrence of uniform cross-sectional areas in muscle, and the optimal measurement points within the cross-sectional area of muscle have not been thoroughly investigated.

The RF is the only pennate muscle in the quadriceps femoris muscle group that comprises muscle fibers running laterally and medially from the central aponeurosis (CA) located in the middle of the muscle. The CA originates from the RF, extends distally for one-third of the muscle length, and is particularly visible in the central part of the RF. Owing to its unique shape and continuity [8], as well as its deformation during muscle contraction [9], the CA is commonly used as a landmark for identifying the RF in imaging studies [10].

This study aimed to evaluate the non-uniformity of the cross-sectional area of atrophic muscle using the CA as a landmark for the center of the RF.

## Materials and methods

The study participants were individuals who were admitted to our hospital for transcatheter aortic valve replacement (TAVI) or elective open-heart surgery and had not previously undergone cardiac rehabilitation. The study was conducted in accordance with the Declaration of Helsinki and the Ethical Guidelines for Life Science and Medical Research Involving Human Subjects. The study protocol was approved by the Ethics Committee of Akita University School of Medicine (approval number: 2982). Written informed consent was obtained from all study participants after providing them with adequate information about the study.

Imaging of the short-axis view of the mid-thigh

The short-axis view of the mid-thigh was imaged with the patient in the supine position at rest. A pocket-sized ultrasound diagnostic device (Pocket Echo Miruco, Sigmax, Tokyo, Japan) with a 5-cm-wide linear probe (10 MHz) was used for the measurements. The midpoint of the line connecting the anterior superior iliac spine (ASIS) and patella was marked on the skin surface at the mid-thigh location. The probe was positioned at this site such that its center was at the same location as the marking, and an ultrasonographic image was acquired (these images, acquired using landmarks on the body surface, were denoted as “On Surface” images). The probe was then moved horizontally in the outward and inward directions to position the RF’s CA at the center of the image, and an ultrasonographic image was acquired (these images, acquired using image landmarks, were denoted as “In Images”). If the CA was located in the center of the image during the “On Surface” imaging, a second image was taken, called an “In Images.” Furthermore, the distance (deviation displacement) from the mid-thigh (midpoint of the line connecting the ASIS and patella) to the CA was measured in 0.5 cm increments from the skin surface, marking the CA using a measuring tape. For both “On Surface” and “In Images” measurements, the probe was placed perpendicular to the skin, and after applying a water-soluble gel, images were acquired with minimal tissue compression.

If it was difficult to identify the CA, it was confirmed by checking for continuity by sliding the probe up and down or checking for changes due to muscle contraction.

Measurement of QMT

**Figure 1:**
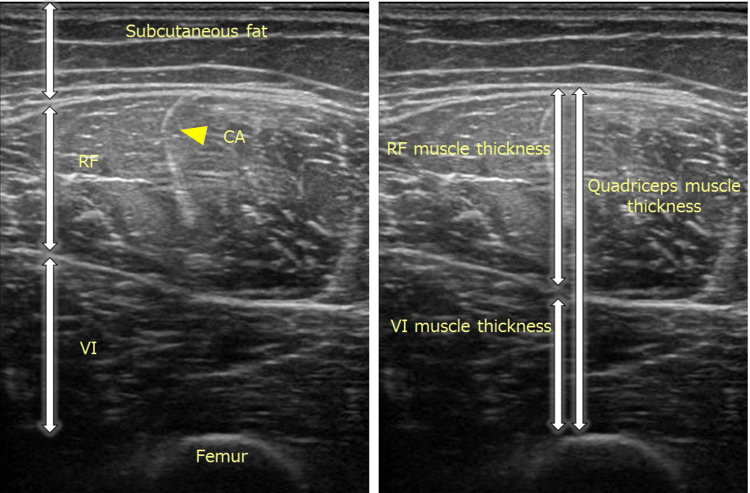
Ultrasound images showing the method for measuring the muscle thickness of the quadriceps femoris. The right panel shows the lateral view, while the left panel presents the medial view. Abbreviations: RF, rectus femoris; VI, vastus intermedius; CA, central aponeurosis

The thickness of the RF, vastus intermedius (VI), and Quad were measured with a precision of 0.1 mm using the ultrasonographic images described earlier (Figure 1). RF thickness was defined as the distance between the superficial and deep fascia; VI thickness was defined as the distance between the superficial fascia and bone directly above the femur; Quad thickness was defined as the distance between the superficial fascia of the RF and directly above the femur. The thickness of each muscle was measured along a line perpendicular to the center of the image. In other words, for “In Images” measurements, the CA served as the starting point for the perpendicular line.

Collection of patient characteristics

Data on basic patient characteristics, including age, sex, diagnosis, height, and weight, were collected from medical records. In addition, grip strength, walking speed, Short Physical Performance Battery (SPPB) score, and thigh circumference were measured at the middle level of the thigh.

Statistical analysis

To confirm the deviation of the RF center, the deviation displacement of the distance (deviation displacement) from the mid-thigh to the CA was divided into four groups: ≤1 cm, 1.5-2 cm, 2.5-3 cm, and ≥3.5 cm. The percentage of each group was then calculated. Since the probe width was generally 3 cm or 5 cm, a deviation of 1.5 cm or 2.5 cm indicated that the CA (at the RF center) did not enter the probe. Additionally, the difference in muscle thickness between “In Images” and “On Surface” measurements was calculated, and 95% confidence intervals (CIs) were calculated for RF, VI, and Quad. To investigate the relationship between the deviation amount and muscle thickness difference, Pearson’s correlation coefficient was calculated. The R software package was used for statistical analyses, and the significance level was set at 5%.

## Results

The study population consisted of 51 consecutively admitted patients (102 limbs), of which 28 (54.9%) had aortic stenosis. The basic characteristics of the study population are summarized in Table 1.

**Table 1:** General characteristics of participants (N = 51). Values are expressed as mean ± SD, median (25th percentile and 75th percentile), or number (%). Abbreviations: BMI, body mass index; SPPB, Short Physical Performance Battery; ASIS, anterior superior iliac spine; SD, standard deviation

Parameters	Unit	Values	Minimum-maximum
Age	Years	78.3 ± 9.3	54-94
Sex (male)	%	20 (37.7)	-
BMI	kg/m^2^	22.8 ± 4.7	2-31.7
Right grip strength	kg	20.0 ± 9.8	7.9-52.1
Left grip strength	kg	17.8 ± 9.7	6.8-51
Gait speed	m/s	0.8 ± 0.3	0.3-1.4
SPPB	Point	9.0 (5, 10.5)	0-12
Right length of the ASIS to the patella	cm	38.8 ± 3.0	32-46
Left length of the ASIS to the patella	cm	38.7 ± 3.0	32-46
Right circumference of mid-thigh	cm	45.0 ± 5.2	36-58
Left circumference of mid-thigh	cm	45.1 ± 5.6	34-58

Assessment of the deviation displacement of the RF center from “On Surface” measurements

**Figure 2:**
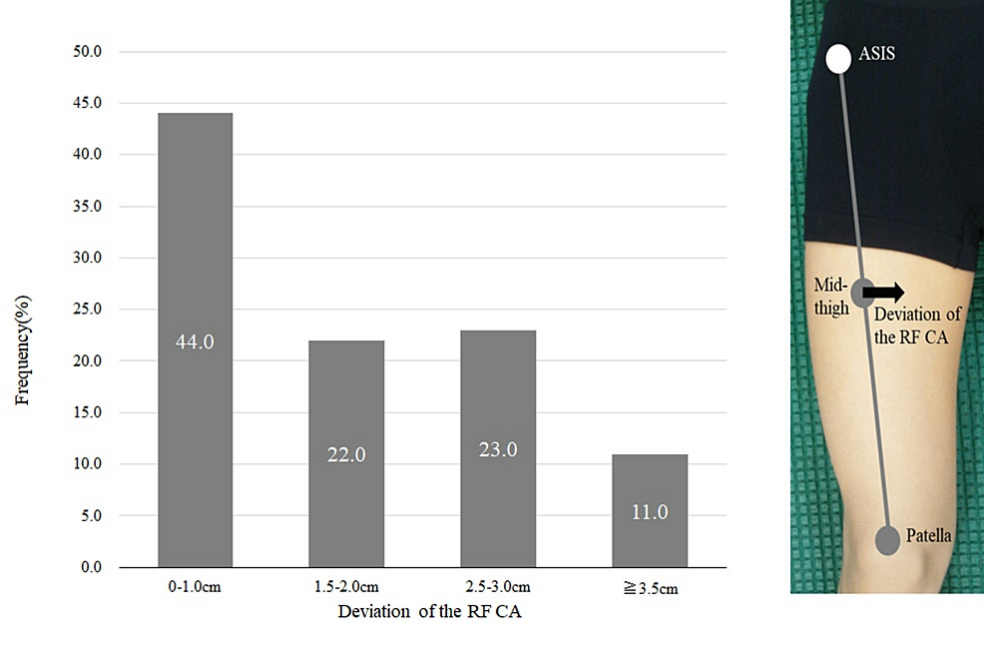
Assessment of the displacement of the central part of the rectus femoris using the “On Surface” measurement. The schematic on the right shows the measurement method, and the graph on the left shows the histogram. In more than half of the cases, the center of the quadriceps femoris muscle is displaced by ≥1.5 cm, and in more than one-third of the cases, it is displaced by ≥2.5 cm. Abbreviations: RF, rectus femoris; CA, central aponeurosis; ASIS, anterior superior iliac spine

**Figure 3:**
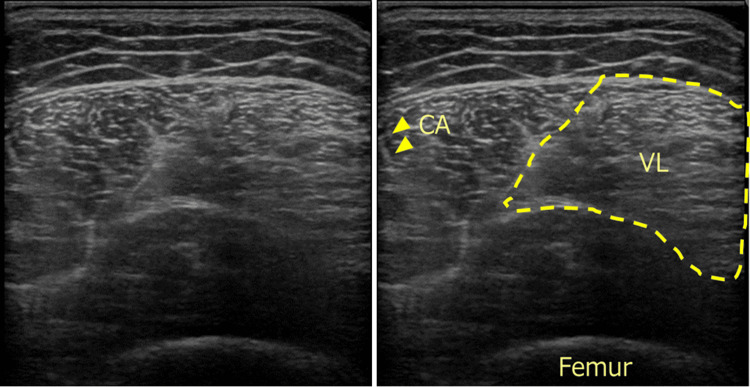
Representative ultrasound image of the rectus femoris. Representative ultrasound image of the rectus femoris showing a deviation of >2.5 cm (right). The guide is shown in the left panel. In cases with deviations of >2.5 cm (33%), the VL occupies half of the image. Abbreviations: CA, central aponeurosis; VL, vastus lateralis

Figure 2 shows the deviation displacement of the central part of the RF from the surface (the center of the thigh). Displacements ≥ 1.5 cm were observed in 56 (54.9%) limbs, and displacements ≥ 2.5 cm were observed in 34 (33%) limbs. Of the displacements, 54 (52.9%) limbs exhibited inward displacement (maximum, 4 cm; minimum, 1.5 cm), while only two (2%) limbs displayed outward displacement (2 cm and 4 cm, respectively). Furthermore, the maximum inward displacement was 4 cm, whereas the maximum outward displacement was 2 cm. Among the cases in which the displacement was ≥2.5 cm, there were instances where the VI was included in the “On Surface” images (Figure 3).

Differences in muscle thickness measurements between “On Surface” and “In Images” readings

Table 2 lists the muscle thicknesses measured using “On Surface” and “In Images.” The RF thickness was significantly lower with “On Surface” than with “In Images” (right, p < 0.001; left, p = 0.007). The VI thickness was not significantly different between the “On Surface” and “In Images” measurements (right, p = 0.46; left, p = 0.31). The Quad thickness was significantly lower with “On Surface” than with “In Images” (right, p = 0.004; left, p < 0.001).

**Table 2:** Muscle thickness of landmarks on the body surface and in the images (N = 102). Values are expressed as mean ± SD (maximum-minimum). Abbreviations: RF, rectus femoris; VI, vastus intermedius; CA, central aponeurosis; SD, standard deviation

	Landmarks			
Muscle thickness	On the body surface		In the images (CA)	
RF muscle				
Right	10.8 ± 2.8 (6-17)		12.0 ± 2.7 (6-18)	
Left	10.4 ± 3.8 (0.5-19)		11.6 ± 2.5 (6-19)	
VI muscle				
Right	8.6 ± 3.0 (3-16)		8.6 ± 3.4 (4-20)	
Left	8.8 ± 3.4 (1-19)		8.4 ± 2.9 (5-19)	
Quadriceps muscle				
Right	20.9 ± 5.5 (10-33)		22.3 ± 5.7 (10-35)	
Left	20.5 ± 5.3 (8-33)		21.9 ± 4.6 (12-35)	

**Figure 4:**
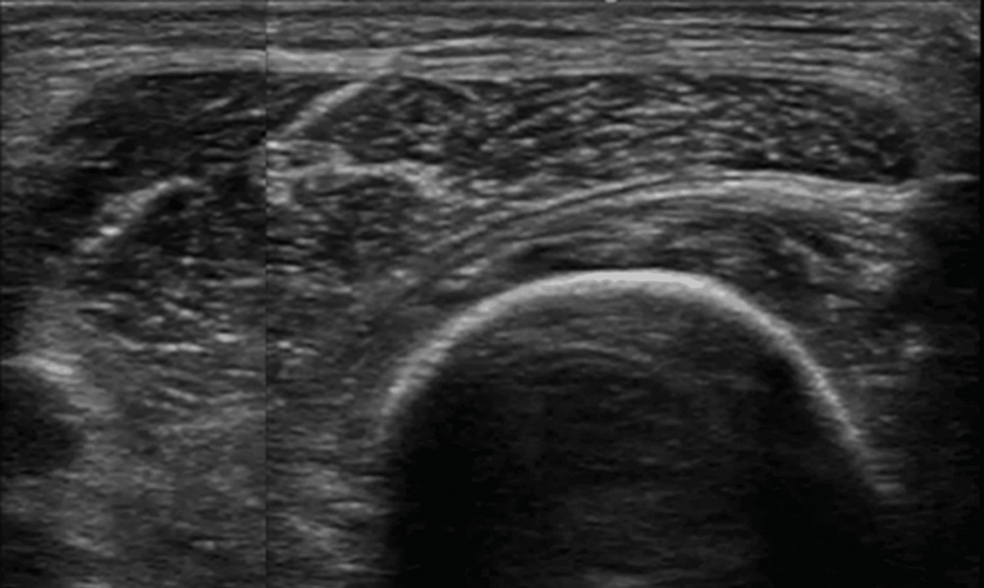
Representative ultrasound image of a non-uniform muscle cross-section of the rectus femoris. The image was created by combining two separate images.

Table 3 shows the differences in muscle thickness between “On Surface” and “In Images” measurements (“In Images” minus “On Surface”). For all muscles, including RF (95% CI: 0.709-1.691; maximum, 10.5 mm/minimum, -7 mm), VI (95% CI: -0.732-0.132; maximum, 7.8 mm/minimum, -14 mm), and Quads (95% CI: 0.87-1.93; maximum, 10 mm/minimum, -6 mm), approximately 50% of the samples showed a difference of ≥1 mm. Figure 4 shows a representative ultrasound image with significant and uneven differences.

**Table 3:** Differences in muscle thickness and frequency (N = 102). Values are expressed as %. Differences were calculated by subtracting “On Surface” values from “In Images” values. Abbreviations: RF, rectus femoris; VI, vastus intermedius; Quad, quadriceps femoris; “On Surface,” landmarks on the body surface; “In Images,” landmarks

	≦3 mm	-1--2 mm	0 mm	1-2 mm	3-4 mm	≧5 mm
RF	1	9.2	49	17.3	12.2	11.2
VI	7.6	21	55.2	11.4	2.9	1.9
Quad	2	7.1	50	14.3	14.3	12.2

Correlation between displacement and differences in muscle thickness

**Figure 5:**
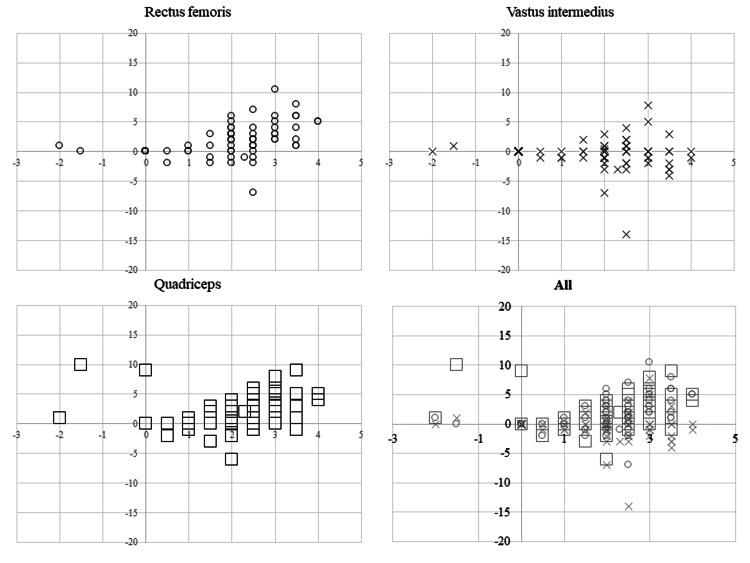
Scatterplots of displacement and muscle thickness differences. In all four graphs, the y-axis indicates differences in muscle thickness (mm), while the x-axis indicates displacement. The rectus femoris is represented by white circles, the vastus intermedius by crosses, and the quadriceps femoris by white squares. The lower left graph shows a superposition of all the graphs.

A significant positive correlation was observed between deviation displacement and the difference in muscle thickness for the RF (r = 0.551, 95% CI: 0.393-0.677, p < 0.001). No significant correlation was observed between the amount of deviation and the difference in muscle thickness for the VI (r = -0.105, 95% CI: -0.3-0.099, p = 0.313). A significant positive correlation was observed between the amount of deviation and the difference in muscle thickness for the Quad (r = 0.381, 95% CI: 0.195-0.54, p < 0.001). All scatterplots are shown in Figure 5.

## Discussion

In this study, our primary focus was to use ultrasonography to investigate the morphological changes occurring in atrophied muscle. We used the CA of the RF as a reliable landmark to assess these changes. We enrolled patients who were to undergo elective cardiac surgery (TAVI or open-heart surgery), had not undergone rehabilitation, and were expected to have muscle atrophy due to physical activity restrictions. The hand grip strength, gait speed, and SPPB score in this study were similar to those reported by Saitoh et al. in patients who underwent preoperative TAVI [11]. These values correspond to sarcopenia [12] and frailty [13]. The representative measurements of grip strength, walking speed, and SPPB scores showed consistently low values, collectively suggesting that our sample could be considered representative of the muscle atrophy model.

The RF is often used as a representative ultrasonographic indicator of muscle thickness [2]. Previous studies measured muscle thickness solely as the space between the superficial and deep aponeuroses, as per ultrasonographic images acquired at measurement points determined by surface morphology landmarks [2,14]. However, in this study, more than half of the atrophied muscles deviated by >1.5 cm from the measurement points on the body surface, and the muscles were found to be thicker at the central part of the muscle cross-section than at the edge. Deviations > 2.5 cm were observed in approximately 30% of the cases, and even with a 5 cm probe, measurements were taken at the edge of the muscle cross-section, possibly resulting in thinner measurements. Previous studies have shown differences in ICC between healthy individuals and critically ill patients [6,7], possibly due to the non-uniformity of muscle morphology in atrophied muscles. Furthermore, without using landmarks on images, it is possible to mistakenly measure the lateral vastus. Thus, these findings suggest that the non-uniformity of muscle morphology in atrophied muscles may be a factor contributing to measurement errors and that landmarks within the body are crucial for the accurate measurement of muscle thickness.

However, for the VI, there was no significant difference in muscle thickness between the two methods, and no correlation was observed between the deviation displacement and the difference in muscle thickness. The difference in VI muscle thickness also showed an opposite trend to that of the RF. Considering that the VI is attached to the femur, it is reasonable to assume that the RF is displaced within the VI. Therefore, the results for the Quad reflect the influence of the RF. In other words, when measuring the thickness of the quadriceps muscle using ultrasonography, landmarks within the body should be based on the RF, where the CA of the RF should be considered as a good landmark.

Limitations and future directions

In this study, the CA was used as an index for the central portion of the RF, and non-uniformity of the cross-sectional area in the atrophied muscles was confirmed. However, further investigations are needed to evaluate the reliability of the CA as a landmark for examination. Moreover, to our knowledge, this study is the first to reveal the displacement of the RF. Subsequent studies need to explore the reversibility of this displacement and its potential relationship with the physical activity level of affected individuals.

This study has several limitations. This was a cross-sectional study, and whether the observed non-uniformity in the muscle cross-section is truly derived from low physical activity-induced muscle atrophy is unclear. Additionally, muscle thickness was measured only at the midpoint between the ASIS and the upper edge of the patella. However, several measurement points for muscle thickness in the Quad have been reported [2], and it remains unclear whether similar differences in muscle thickness occur at these other points, particularly those in the distal two-thirds of the muscle.

## Conclusions

In over half of the patients undergoing elective cardiac surgery, the central portion of the RF exhibited medial displacement, and the thickness of the displaced area was thinner than that of the central portion, showing non-uniformity. To identify the same location in repeated longitudinal measurements, landmarks in ultrasonographic images are necessary, and the CA of the RF may be useful for this.
